# Effects of Covid-19 on the elective surgery: an approach based on the case of the Italian Province of Bolzano

**DOI:** 10.1007/s43999-023-00021-x

**Published:** 2023-03-15

**Authors:** Mirko Bonetti, Carla Melani

**Affiliations:** Observatory for Health – Health Department Province of Bolzano, Bolzano, Italy

**Keywords:** Systematic component of variation, Small Area variation analysis, Elective procedures, COVID-19 impact, Declaration of conflicting interests, The authors declared no potential conflicts of interest with respect to the research, Authorship, And/or publication of this article

## Abstract

**Background:**

As of February 2020, the rise of COVID-19 cases led to significant pressures in the Northern parts of Italy, including the province of Bolzano (a small territorial reality on the border between Italy and Austria), and left the Italian National Health System (NHS) unprepared for the initial wave of the SARS-CoV-2 pandemic.

**Objective/Methods:**

By dividing the analyses into two phases, the study assesses the effect of COVID-19 for the years 2018–2019 and 2020–2021. The first phase highlights the hospitalization rates in the Province of Bolzano in comparison to other Italian regions. In the second step, the Systematic Component of Variation (SCV) has been applied to calculate the differences between the admission rates for the elective surgery (tonsillectomy, vein stripping, hip replacement, knee replacement, and arthroscopy) in the four health districts (HDs) of the Province of Bolzano.

**Results:**

Overall, the findings demonstrate that the effect of COVID-19 cases has resulted in a 20% reduction in hospitalization rates. A variation of less than 30% for knee and hip replacements and up to 75% for vein stripping is seen for elective surgeries. According to the SCV values for each elective procedure, the results indicate comparable levels of variation across the two time periods, with tonsillectomy showing the lowest levels of variation, arthroscopy and vein stripping having the highest levels, hip replacement having a high level and knee replacement having a low-medium level.

**Conclusions:**

The data show no significant changes in the variation between the four HDs in the province of Bolzano, suggesting that the COVID-19 cases have a proportionate impact on hospitalization rates.

## Introduction

The autonomous province of Bolzano, located on the border between Italy and Austria, is a modest territorial reality, with a population of over 500,000 inhabitants and a mountainous territory of 7,400 km^2^. Since 2006, one Local Health Authority (LHA) in the local government of Bolzano coordinates four Health Districts (HDs), namely: Bressanone covering 14% of the entire population, Brunico covering 15%, Merano covering 26% and Bolzano that covers 45% of the population. The Province of Bolzano is home to seven public and six private hospitals. The HDs Merano, Bressanone and Brunico each have two public hospitals, while the city of Bolzano has one significant public hospital. In contrast, there are two private hospitals in each of the HDs Bolzano, Merano, and Bressanone. Private hospitals have played a larger role in recent years, but their figures indicate that they only account for a small portion of all acute discharges (from 3% in 2018 to 7% in 2021). Due to the proliferation of COVID-19 cases in Italy’s northern areas, including the Province of Bolzano, the Italian National Health System (NHS) was caught unprepared by the first wave of the SARS-CoV-2 pandemic at the end of February 2020. The analyzed periods can be divided into four waves based on the prevalence rates per 100,000 people. The province of Bolzano displayed higher values than the national level in the first wave (from the end of February 2020 to the end of May 2020), particularly in April 2020 (300 approx. vs. 180 approx.), which in the post-first wave period (during the summer months) have been fixed at 40 for both values. Bolzano’s rate increased more quickly during the second wave beginning in October 2020, with notable disparities in the period between November and December 2020 (over 2,000 vs. 1,300 approx.). The rate for the province of Bolzano then climbed during the third wave in January–February 2021, exceeding the national average (about 2,600 vs. 800), before declining in March 2021 (700 vs. 900 approx.). The national value has always been greater after the third wave (from April to October 2021), especially between April and May (200 vs. 600 approx.), and both rates reached a similar level in September (200 approx.) and October (150 approx.). Bolzano’s rate resumed to expanding faster than the national average from November through mid-December 2021 (1,200 vs. 500), determining the fourth wave [1]. The NHS ensured the reorganization of hospitals as a result of the emergency faced at both the territorial and hospital levels by raising the number of Intensive Care Unit (ICU) beds and reducing the number of less severe COVID-19 patients in the regular wards [2, 3]. In these conditions, there were fewer admissions, mostly for elective surgery and non-essential medical care. However, data revealed that admissions for serious medical conditions also decreased (e.g., stroke, acute myocardial infarction) [4]. The pandemic's widespread uncertainty, the fear of spreading the disease, and preventative restraining measures are some of the reasons for the reduction in admissions [5, 6]. As a result, patients made decisions to postpone or avoid hospitalization for non-emergency surgical treatments and elective operations in coordination with their doctors [7]. As reported by Italian sources of data collected prior to the COVID-19 pandemic's growth, a comparison of the four HDs’ hospitalization rates to the national average reveals an internal variation. The study uses the Small Area Variation Analysis (SAVA) to demonstrate how the hospitalization rates differ, using the geographic variation in healthcare within the HDs as a point of comparison [8]. This approach is crucial for health planning because it confirms the existence or absence of geographic variations within the same region and examines a variety of factors that are intended to determine how hospitalization rates are distributed and predict any increase in spending and in the incurred costs. The NHS benefits from the identification of geographic variations within the same territory since it makes resource planning easier and fosters integration both horizontally (using the same resources for the same needs) and vertically (different resources for different needs). Based on the correlation between activity volumes and outcomes, the literature assigns reference values for specific surgical operations (such as those in the oncological, orthopedic, and cardiological areas). There is an unwarranted variation when the health system has a tendency to diverge internally, which indicates the existence of an internal variability and suggests that it may not be able to respond to patients' demands equally [9]. When this occurs, policymakers have to undertake concrete measures that lessen the unwarranted variation while simultaneously have to monitor those elective treatments that don't have reference standards. The rates of these operations are typically determined at the doctor's discretion instead of taking into account the needs and preferences of the patient. This fact results in the possibility of potential variations occurring within the same territory. Therefore, by assuming that the system is unable to address the patient's demands, it is crucial to study and quantify the unwarranted variation since it affects all-equal access to services, the use of resources, and the outcomes. Understanding how to identify and calculate unwarranted variation is helpful since it appears that managing it is necessary for the NHS to ensure the quality of the services provided in hospitals. According to numerous studies, the decisions taken by the health system at the level of local governance (LHAs), whose evaluation is crucial to raising the quality of their services, also have an impact on the geographical diversity in hospitalization rates. The health system's discretion affects hospitalization rates in two ways: whether the aim for specific clinical setting or whether they aim for precise clinical care guidelines. Calculating a random variable and a variable assigned to the data accuracy is essential for the analysis of the causes of variation at the level of supply and demand for health services. Clinical variables and high-quality coding diagnoses are two elements that affect the final results. The demand is related to choices made by doctors and patients depending on their socio-health characteristics and lifestyles. On the other hand, the supply is linked to the clinical approaches as they are expressed through either specific recommendations or decisions taken at the governance level. The resources available have an impact on the supply as well (e.g., number of beds, number of specialists, waiting time, availability of operating rooms, private financing of health organizations, incentives for professionals). This can be taken into consideration for the evaluation of physicians' decisions regarding their patients in two ways: first, by analyzing the factors that combine the available resources with clinical indications, and second, by giving weight to the interactions between factors that apply respective to the supply and demand [10, 11]. The purpose of this article was to explore the geographical variation in hospitalization rates for a number of elective surgery procedures (tonsillectomy, vein stripping, hip replacement, knee replacement, and arthroscopy) across the HDs in the autonomous province of Bolzano. The study focuses on any specified elective operations that call for the reference standards in order to instruct policymakers in assigning measures and interventions that reduce the unwarranted variation at minimum. The objective was to show if a small territorial reality, like the Province of Bolzano, measures a minimal variation before determining whether the variation is unwarranted [12]. The study defends the idea that the differences in hospitalization rates based on geographic variation in the four HDs of the province of Bolzano (with the subsequent risk of pertinent variations within the same territory), reflect the physicians' discretionary decisions about the access of patients to healthcare rather than the needs and preferences of the patients. In order to achieve this goal, the study examines the potential COVID-19 impact for the years 2018–2019 and 2020–2021. The results demonstrate that the hospitalization rates vary, either as a result of variation in the level of data accuracy used for the individual HDs or as a result of differences related to geographical features and local health care systems.

## Methods

Because there is no standard reference value for elective surgery, the use of a technique based on a temporal comparison of hospitalization rates throughout the years has been deemed overly reductive. For this reason, it is crucial to compare similar realities in order to comprehend the position of the observed area. As of 2010 the province of Bolzano takes part to the Italian Regional Performance Evaluation System (IRPES) developed by Mes-Lab-Institute of Management of Sant’Anna School of Advanced Studies (Pisa, Italy), which is a system that measures the multidimensional performance of each healthcare organization with over 400 indicators. It is worth noticing that in the project the indicators have been calculated at the regional level (both Local Health Authorities and teaching hospitals), covering regions of Apulia, Bolzano, Friuli-Venezia Giulia, Liguria, Lombardy, Marche, Umbria, Piemonte, Trento, Tuscany and Veneto [13]. For each observed procedure (tonsillectomy, hip replacement, knee replacement, vein stripping, and knee arthroscopy), the inclusion criteria have been extracted from this project based on the International Classification of Diseases, 9th revision, Clinical Modification Codes (ICD9CM) (Table 1).

**Table 1:** Inclusion criteria for the observed elective procedures

Procedure	Exclusion criteria (Diagnosis)	ICD-9-CM Codes
Tonsillectomy (≤ 17 years)	Neoplasm, trauma, poisoning	28.2 or 28.3
Hip replacement (≥ 65 years)	Fracture of neck of femur	81.51 or 81.52
Knee replacement	Trauma	81.54
Vein stripping		38.59
Knee arthroscopy		80.26

Source: https://performance.santannapisa.it/pes/start/start.php

The Hospital Discharge Administrative Databases provide each hospital with the necessary information regarding the admission in terms of demographic and clinical factors. In the analysis were only included the admissions to public hospitals for patients who were residents from Bolzano province based on their discharge dates between 2018 and 2021. The study did not take into account private hospital admissions since these are not included by the NHS in the health planning because they are paid for by the patients themselves. They are few in number for many more. First, the study examined statistical data from four HDs in the Bolzano province and other Italian regions by factoring standard hospitalization rates (direct method) based on age and gender for each chosen elective surgery. The range, which is the difference between the highest and lowest values, the extreme quotient, which is the ratio of the highest to the lowest value, the standard deviation, which calculates how widely the data are dispersed from the mean, and the coefficient of variation, which is the ratio of standard deviation to the mean, have all been used to measure the degree of variation between the admission rates in the four HDs, which can be intuitively and mathematically more or less complex. The most practical option is the systematic component of variation (SCV), as this technique may be used to create reliable measures of hospitalization variation. More specifically, this method calculates the data variation based on the number of observed hospital admissions in comparison to the expected hospitalization rates, taking into account the population's age and gender distribution. The following calculation formula:

$$SCV=\frac1k\left(\sum\frac{\left(y_i-e_i\right)^2}{e_i^2}-\sum\frac1{e_i}\right)$$

Where (*k*) describes the number of observed areas, (*y*_*i*_) displays the observed events and (*e*_*i*_) displays the expected occurrences in the patient's area *i.* The SCV has as usual been raised by 100. Age and gender are taken into account in the standardized hospitalization rate to determine the number of anticipated incidents (indirect method, admission rates in the total population as the standard). Despite determining the accuracy of its estimation, the SCV's value is unaffected by the number of areas that were examined. This value is thought to be sensitive to the population density in each area. The data can thus be modified to reflect the internal variability of each location. SCV values less than 1 indicate a level of no variation, with levels of minimal variation between 1 and 3, medium variation between 3 and 6, high variation between 6 and 10, and extremely high variation above 10 [14–16]. If this value is expanded, it implies that the physician's discretionary choice takes precedence, as influenced by various factors (different clinical approaches, more availability in terms of beds and operating rooms, shorter waiting times, different resources available), over the needs and priorities of patients, unless there are different care settings for the same procedure (day hospital or ambulatory care). The analysis is split into two parts in the study: comparing hospitalization rates in the province of Bolzano to those in other Italian areas and calculating the SCV in part two. The targets of the SCV calculation for each elective procedure examined were the differences resulting from the influence of the COVID-19 pandemic throughout two periods, from 2018 to 2019 (before COVID-19), and from 2020 to 2021 (after COVID-19).

## Results

The overall hospitalization rates have lessened about the 20% because of the waves of Covid-19’cases, whereas the studied elective procedure showed variations that approach from less than 30% for the knee and hip replacement to 75% for the vein stripping. But if we set 100 the average IRPES regions, the findings show that the Province of Bolzano in both periods had higher rates as related to the regional average, except for the vein stripping (Table 2).

**Table 2:** Overall hospitalization rate and for elective procedures observed per 1,000 population (2018–19 vs 2020–21)

Procedure	2018–19	2018–19 (mean regions = 100)	2020–21	2020–21 (mean regions = 100)
Overall	135.62	113.41	107.34	117.19
Tonsillectomy (≤ 17 years)	3.16	139.67	1.35	128.79
Hip replacement (≥ 65 years)	4.91	148.27	3.60	132.59
Knee replacement	1.71	120.91	1.24	115.46
Vein stripping	0.36	97.46	0.09	55.49
Knee arthroscopy	2.55	183.75	1.66	187.19

Source: Elaborations from https://performance.santannapisa.it/pes/start/start.php

Even though hospitalization rates have decreased, there is still a substantial variation across the four HDs in the time after the COVID-19 impact. The HDs Bressanone and Brunico showed higher rates (over 160 in 2018–2019 and approximately 140 in 2020–2021) in both time periods in comparison to the HDs Bolzano and Merano (about 150 in 2018–2019 and 120 in 2020–2021). More than the other HDs which dropped the rates less than 20%, the HD Bolzano saw a 27% decrease in hospitalization rates. This data suggests that the leading hospital in Bolzano encountered a considerable number of COVID-19 cases, which had an adverse effect on the other pathologies that were less time-sensitive.

### Vein stripping

The four HDs displayed similar trends regarding the initial variations as the hospitalization rates for vein stripping decreased (over 70% in the HDs), with the HD Brunico registering the highest value (137.22 in 2018–2019 and 36.29 in 2020–2021), as opposed to the other HDs that displayed a minimal value. Overall, it can be concluded that the SCV value grew during the 2020–2021 period from 216.68 to 280.75, demonstrating a very high variety (Table 3). In 2020–2021, there are 74 interventions as opposed to 306 in the two years prior (- 76%). 90% of the populations of the HDs Bressanone and Brunico have undergone surgery in their home district hospitals. But for those living in the HDs Bolzano and Merano because this treatment was carried out in an outpatient setting, there are less disparities across the districts overall, indicating a transition from a very low variation (3.23 in 2018–2019) to a very high variation (18.93 in 2020–2021).

### Tonsillectomy

The hospitalization rate has dropped for tonsillectomy procedures; the HD Merano exhibited a shift in variation from 406.73 in 2018–2019 to 138.04 in 2020–2021 (-66%). The other HDs cut their rates by 50%, demonstrating that the SCV is at a lesser level (Table 3). In the following two years, the number of interventions fell by more than half (-57%), from 633 (in 2018–2019) to 270 (in 2020–2021) (Table 3). 100% of the HD Bressanone inhabitants received medical care in home district hospitals. Both hospitals of the HD Bressanone have offered medical treatment also for the residents of the HD Brunico, whose hospitals do not provide these specific surgical interventions. The HD Merano's population experiences the similar problem, albeit with lower values (about 90%). The hospitals in the other HDs received patients from more than a third of the HD Bolzano for surgical procedures.

### Hip replacement

The SCV value for hip replacement surgery has reduced, confirming the wide variation, with the two HDs with the highest rates (about 700 in 2018–2019) showing varying rate reductions, with the HD Brunico showing a higher reduction (-36%) and the HD Bressanone a lower reduction (-20%) (Table 3). Over the next two years, there was a 25% decrease in hip replacement surgeries, from 1,016 in 2018–2019 to 759 in 2020–21. The hospitals in the HDs Bressanone and Brunico, which also had to provide for their own inhabitants' requirements, provided surgical intervention for this treatment for 90% of the population of the HD Bolzano. In the HD Merano, 50% of the residents had the medical intervention for this procedure in the HD’s hospitals.

### Knee replacement

For the knee replacement treatment, the SCV value has increased, while it is still at a low-medium level. The HDs Bressanone and Brunico, which had higher rates, demonstrated a rate drop in comparison to the other two districts (30% vs 24%) (Table 3). Compared to 1,466 interventions the year before (in 2018–2019), about 1,073 interventions were made the following years (2020–2021). Regarding knee prosthesis, the HDs Bressanone and Brunico had guaranteed this surgical intervention for the majority of their residents as well as those of the other two districts, in particular for the patients of HD Bolzano.

### Knee arthroscopy

The SCV value has grown by almost three points for the knee arthroscopic surgery, standing on the level of a very high variation and displaying a reduction rate that ranges from 30% for the HD Bressanone (393.58 vs. 275.47) up to 39% for the HD Brunico (443.39 vs. 271.9) (Table 3). According to the data, there were 1,435 procedures for this treatment in 2020–2021 compared to 2,185 in 2018–2019. The hospitals in the HDs Bressanone and Brunico provided this type of procedure to the majority of their own residents (90%), to residents in the HD Merano (approximately 50%), and to the majority of residents in the HD Bolzano (90%).

**Table 3:** Hospitalization rate (per 100,000 population) and Systematic Component of Variation (SCV) for the elective procedure observed (2018–19 vs 2020–21)

	2018-19						2020 - 21					
Procedure	Overall	HD Bolzano	HD Merano	HD Bressanone	HD Brunico	SCV (IC95%)	Overall	HD Bolzano	HD Merano	HD Bressanone	HD Brunico	SCV (IC95%)
Tonsillectomy (≤ 17 years)	315.72	271.10	406.73	333.57	269.57	2.41 (2.31 - 2.51)	134.67	115.86	138.04	167.06	147.29	0.43 (0.39 - 0.46)
Hip replacement (≥ 65 years)	491.35	409.30	433.36	696.31	718.06	9.15 (8.89 - 9.42)	359.37	286.67	345.81	534.76	463.72	8.42 (8.14 - 8.70)
Knee replacement	171.10	147.82	172.21	198.31	220.08	2.79 (2.69 - 2.89)	123.75	109.02	114.73	147.69	164.71	3.68 (3.54 - 3.82)
Vein stripping	35.71	13.79	12.47	43.24	137.22	216.68 (203.79 - 229.61)	8.53	3.32	2.64	6.48	36.29	280.75 (261.94 - 299.46)
Knee arthroscopy	255.02	224.03	137.54	393.58	443.39	23.56 (22.94 - 24.18)	165.50	134.69	94.65	275.47	271.4	26.17 (23.59 - 24.62)

Source: Elaborations from https://performance.santannapisa.it/pes/start/start.php

## Discussion

Prior to the COVID-19 pandemic, data revealed that the province of Bolzano had higher hospitalization rates for several elective surgery diseases than the other Italian areas (despite being a modest Italian region with peculiar territorial and cultural characteristics). The study looks at how the NHS handles patients' access to elective operations that are impacted by unwarranted variation within the same region. The study's primary objective is to determine whether admissions to seven public hospitals in the province of Bolzano ensure an appropriate balance between the patients' needs and preferences and the physicians' discretionary decisions. In order to address the disparities between its four HDs, it appears essential for the NHS to equal needs of all patients. In general, physicians' decision is frequently used to determine whether a patient should be hospitalized for these treatments rather than taking into account their needs. Pointing in that way, the study has examined the elements that contributed to the unwarranted variation for these particular procedures: hip and knee replacement, knee arthroscopy, stripping, and tonsillectomy. The population distribution between the local public hospitals is among factors that have been considered. Thus, the data demonstrate that there is a geographical variation among the four HDs in the province of Bolzano, where the main hospital in Bolzano serves around 50% of the population while the remaining 50% is served by the other six public hospitals. Divergences in waiting times, bed availability, and operating room availability between the seven hospitals are further factors that have contributed to the varied hospitalization rates. Furthermore, other elements like the available financial resources are thought to be important, where Bolzano is known for having the highest health expenditures per capita in Italy, at over 2,500 Euros. As a result, hospitals outside the Bolzano area experience excessive hospitalization rates, which necessitate greater involvement from the community health system in order to stabilize these rates according to hospital response. In light of the aforementioned, the admissions decisions for elective treatments can be assumed to reflect more the doctors' discretion than the needs of the patients, which have been impacted by the unwarranted variation. The analyses, which looked at the SCV values for each elective procedure, revealed proportionate levels of variation between the two time periods, with very high values for arthroscopy and stripping, high values for hip replacement, low to medium values for knee replacement, and low values for tonsillectomy. Relevant causes for the vein stripping variation include the gradual shift from hospital to ambulatory care, more accessibility to surgical procedures, more beds in smaller hospitals, shorter waiting periods, and fewer discrepancies between ambulatory and hospital care. Important elements in the knee arthroscopy variation include lower wait times and access to operating rooms. Hip and knee replacements can be attributed to patient age beyond 65, obesity, inactivity, smoking, and accidents, among other pertinent factors. The HDs Bressanone and Brunico have higher hospitalization rates. The clinical recommendations used in these areas for these surgeries are comparable to those used in Austria, Germany, and other Northern European nations, which is one of the causes of variation. Given that certain doctors have graduated from the medical schools of various nations due to their proximity geographically, it is understandable why there are similarities. The financial accessibility of resources for the procurement of medical equipment and the accessibility of operating rooms are additional essential factors for the high rates in the two areas. As a result, hospitals in the two HDs provide for both their own patients' needs as well as those of patients from neighboring districts. Due to this, the hospitalization rates in these two HDs are higher than the Italian average and comparable to those of Austria, Germany, and Switzerland, where the rates for hip and knee replacements, are 300 per 100,000 and 200 per 100,000 respectively. According to data, the revision rate at 1 year for hip replacement is to 2.0% (compared to 3.3% for Austria) while the revision rate at 1 year for knee replacement is to 1.1% (compared to 1.7% for Germany) [17]. Significant aspects for tonsillectomy include doctors' decision in selecting the criteria for cases to be subjected to surgery admission rates (the eligibility criteria that apply for admission of patients for surgical intervention are more restricted, resulting in lower admission rates for this type of procedure). In light of the study's findings, it can be said that many non-COVID procedures, particularly those that were routinely postponed, have a reduced hospital admission rate in the Province of Bolzano as well as in the other Italian areas. These findings showed a substantial variation in values between the four HDs in the province of Bolzano and higher values in comparison to the national average rates for all of Italy. In the four HDs in the province of Bolzano, the statistics demonstrate no significant changes in the variation during the time after COVID-19. This information points to a similar effect of COVID-19 cases on hospitalization rates. The LHA's next goal is to complete the delayed procedures brought on by the large volume of COVID-19 cases in order to maintain rates at a comparable level among HDs.
